# Supplementary material for: Targeted attenuation of elevated histone marks at SNCA alleviates α‐synuclein in Parkinson's disease
Source: EMBO Mol Med. 2021 Jan 11;13(2):e12188. doi: 10.15252/emmm.202012188 (PMC7863397; doi:10.15252/emmm.202012188)
Supplement: Supplementary file 1 — Appendix [file EMMM-13-e12188-s001.docx]

**Appendix Information**

**Table of Contents**

Appendix Figure S1. Distribution of histone PTM peaks in the regulatory region of human *SNCA*.

Appendix Figure S2. Enrichment of H3K27ac and H3K27me3 at the *SNCA* promoter between control and PD brains.

Appendix Figure S3. Enhancer-associated histone mark H3K27ac in the *SNCA* intron 4 is not significantly different between control and PD brains.

Appendix Figure S4. dCas9-5xGCN4 and scFV-sfGFP-JARID1A form a stable complex.

Appendix Figure S5. Establishment of SH-SY5Y cells stably expressing dCas9-5xGCN4.

Appendix Figure S6. dCas9-5xGCN4 was precisely recruited at the *SNCA* promoter as directed by sgRNAs.

Appendix Figure S7. Immunofluorescence staining and western blot images showing the presence of dCas9 in a representative sgRNA-dCas9 cell line.

Appendix Figure S8. Western blot image showing relatively higher levels of α-SYN expression in SH-SY5Y cells compared to other neuronal cell lines.

Appendix Figure S9. Relative efficiency of sgRNAs in reducing H3K4me3 from the *SNCA* promoter.

Appendix Figure S10. Fluorescence microscopy images showing sfGFP expression from a stable sgA-dCas9-JA cell line.

Appendix Figure S11. Individual components of the CRISPR/dCas9 SunTag-JARID1A system do not affect α-SYN or global H3K4me3 levels.

Appendix Figure S12. Additional gel pictures for Figure 5 and Figure 6.

Appendix Figure S13. Characterization of iPSCs.

Appendix Figure S14. Immunostaining of differentiated sPD iPSCs demonstrates successful differentiation to dopaminergic neurons.

Appendix Figure S15. Sequence of scFV-sfGFP-JARID1A construct in pLvx vector.

Appendix Table S1. The details of postmortem brain tissue samples used in the study.

Appendix Table S2. List of primers used in the study.

Appendix Table S3. List of short guide RNAs used in the study.

Appendix Table S4. Exact p values in each figure.

**Appendix Figure S1. Distribution of histone PTM peaks in the regulatory region of human *SNCA*.**


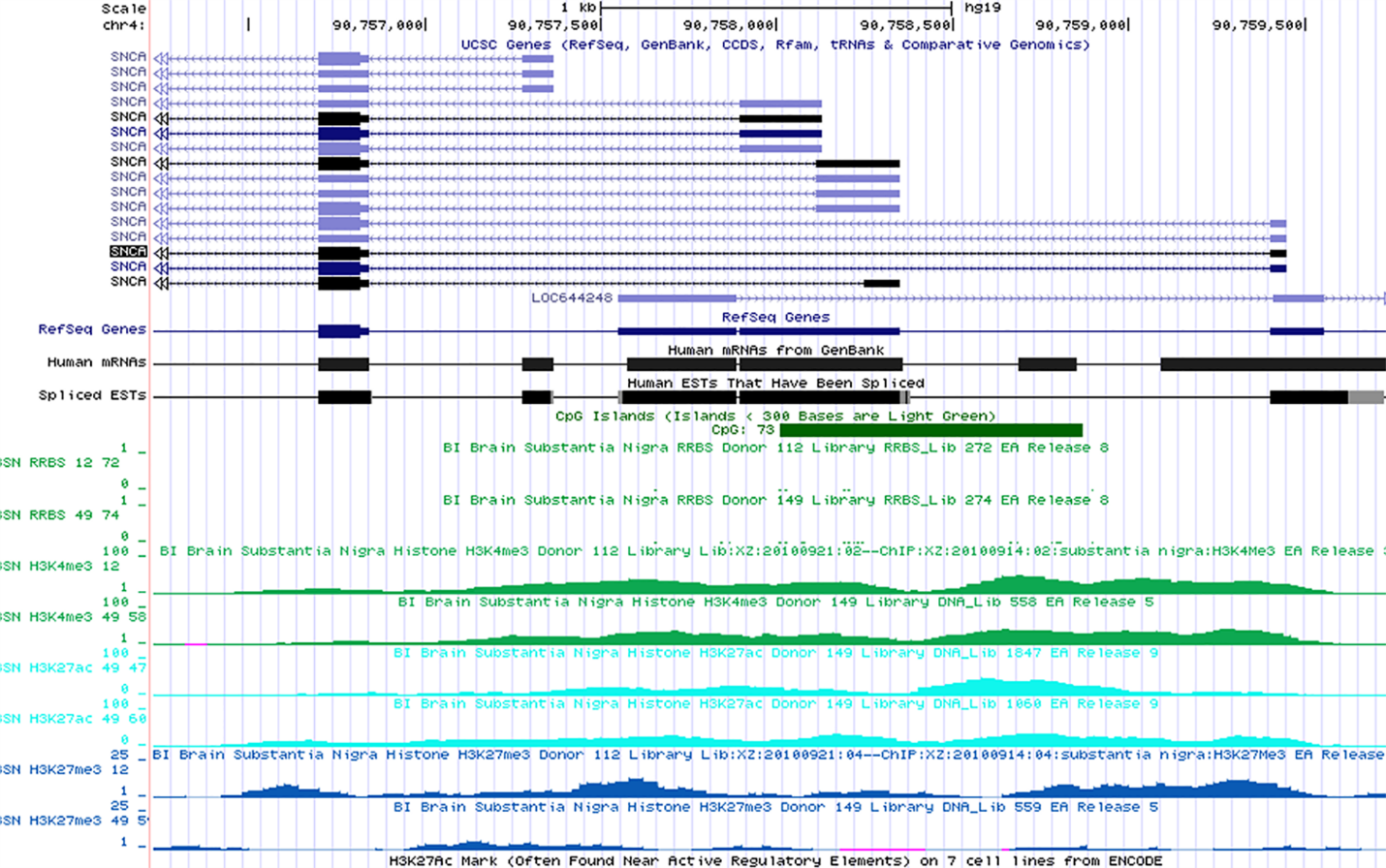


Screenshot from Roadmap Epigenomics Database depicts the distribution of histone PTMs (H3K4me3, H3K27me3, and H3K27ac) at the upstream regulatory region (promoter, its upstream regions, and intron 1) of *SNCA* on chromosome 4 from SN tissues of two healthy adult postmortem brain samples. The green horizontal bar represents the position of the CpG island at the *SNCA* promoter region as per the database (RRBS; reduced representation of bisulphite sequencing). Different transcripts (both coding and non-coding) of *SNCA* with their different TSS are shown by blue horizontal lines where boxes represent exons. The scale for the location of the genomic region from hg19 contig is also shown at the top. The screenshot only displays the genomic region of chromosome 4 from 90,756,000 to 90,760,000 bp. Arrows represent the direction of the gene.

**Appendix Figure S2. Enrichment of H3K27ac and H3K27me3 at the SNCA promoter between control and PD brains.**


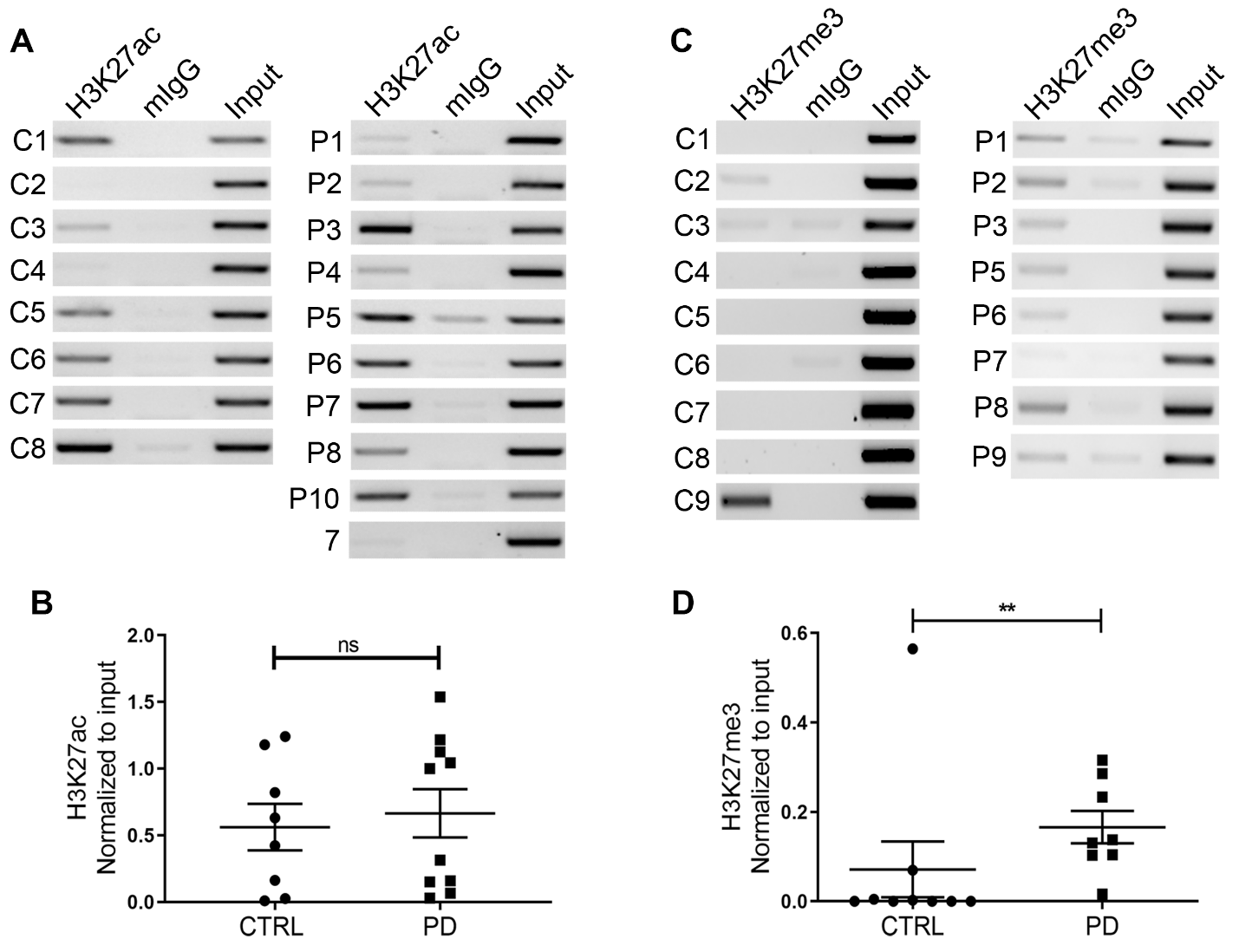


(**A**) Gel images for ChIP showing relative enrichment by H3K27ac at the *SNCA* intron1 from 8 controls and 10 PD brains. (**B**) Graph showing no significant difference in enrichment of H3K27ac at the SNCA promoter between controls and PD subjects. (**C**) Gel images for ChIP demonstrating relative enrichment by H3K27me3 in 9 controls and 8 PD subjects. (**D**) A significantly higher enrichment of H3K27me3 was observed in PD subjects. For both H3K27ac and H3K27me3, PCR was performed for the same region as for H3K4me3. PCR amplified a 188-bp region from the intron 1 of *SNCA* where these marks were found to be enriched in the ENCODE database. Mouse IgG (mIgG) was used as control and the bands were normalized by unbiased amplification from respective inputs.

**P* < 0.05, ***P* < 0.01, ****P* < 0.001, and *****P* < 0.0001, ns = non-significant. Data were analyzed using non-parametric t-test followed by Mann-Whitney post-hoc corrections. Two-tailed p-values were calculated for all.

Data information: Data represent mean ± standard error of the mean.

**Appendix Figure S3. Enhancer-associated histone mark, H3K27ac, in the *SNCA* intron 4 is not significantly different between control and PD brains.**


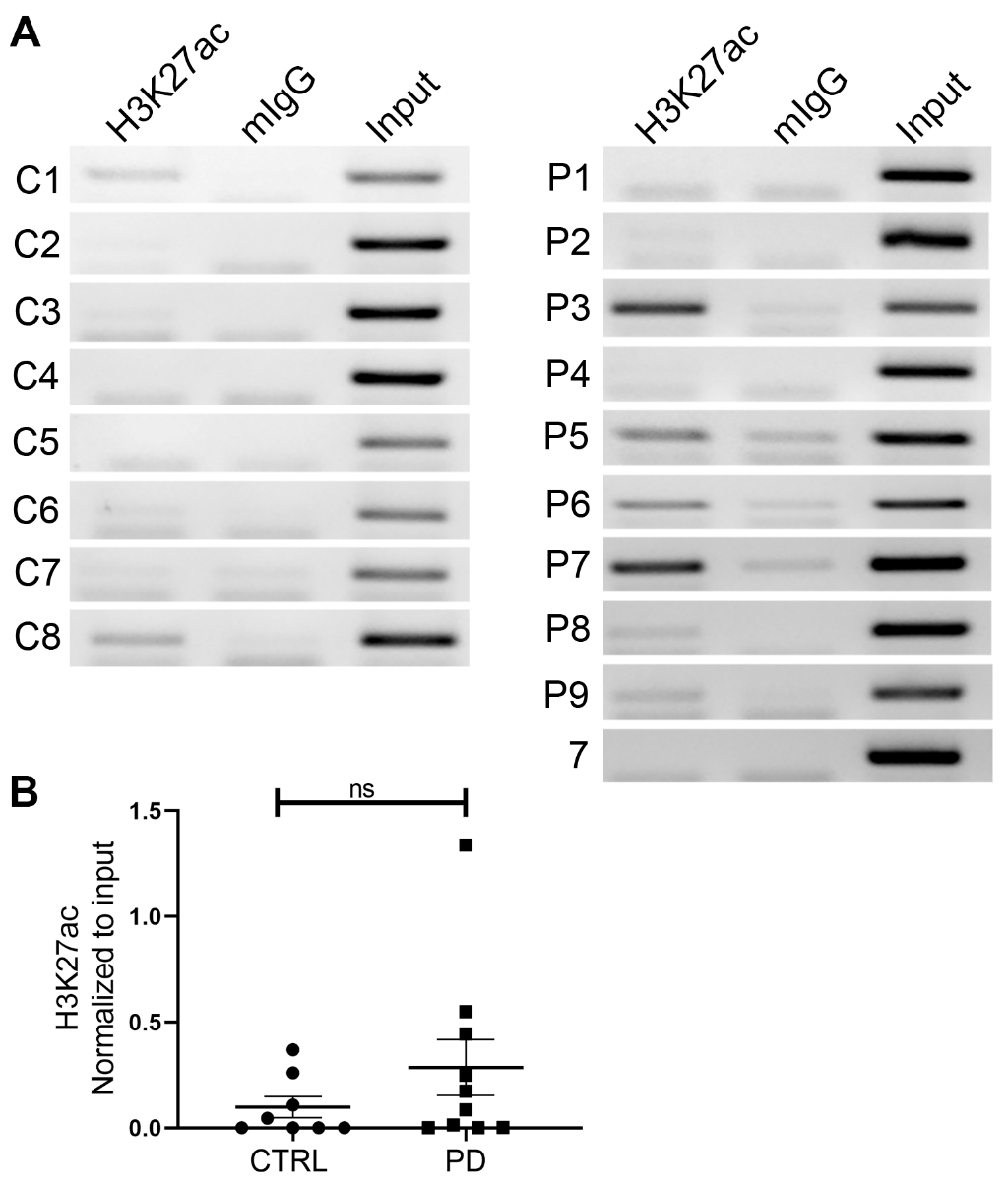


(**A**) Gel images for ChIP showing relative enrichment of H3K27ac in the intron 4 region of *SNCA* in control (n=8) and PD subjects (n=10). The PCR amplified 155-bp region in the intron 4 corresponds to the H3K27ac peak in the ENCODE database. (**B**) Mean of the normalized intensities of relative H3K4me3 enrichment between control and PD were evaluated. No significant difference was observed between the groups. All data are presented as mean ± SEM. ns = non-significant. Data were analyzed using non-parametric t-test followed by Mann-Whitney post-hoc corrections. Two-tailed p-values were calculated.

**Appendix Figure S4. dCas9-5xGCN4 and scFV-sfGFP**-**JARID1A form a stable complex.**


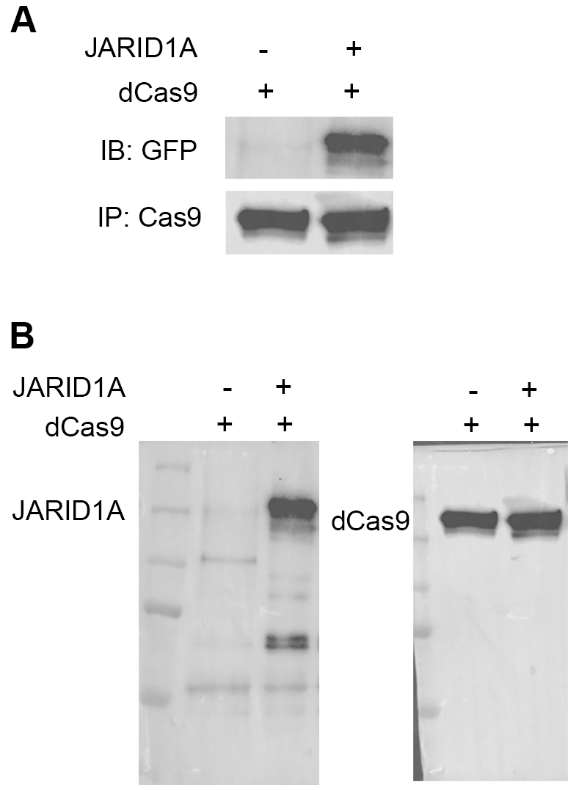


(**A**) dCas9-5xGCN4 plasmid was overexpressed in HEK293T cells with or without scFV-sfGFP-JARID1A. Total protein was isolated and immunoprecipitated with anti-cas9 antibody and immunoblotted against anti-GFP antibody. Cas9 antibody could pull down scFV-sfGFP-JARID1A (~168 kDa) from HEK293T cells expressing both the constructs. (**B**) Full western gel images of immunoblots used in A.

**Appendix Figure S5. Establishment of SH-SY5Y cells stably expressing dCas9-5xGCN4.**

**
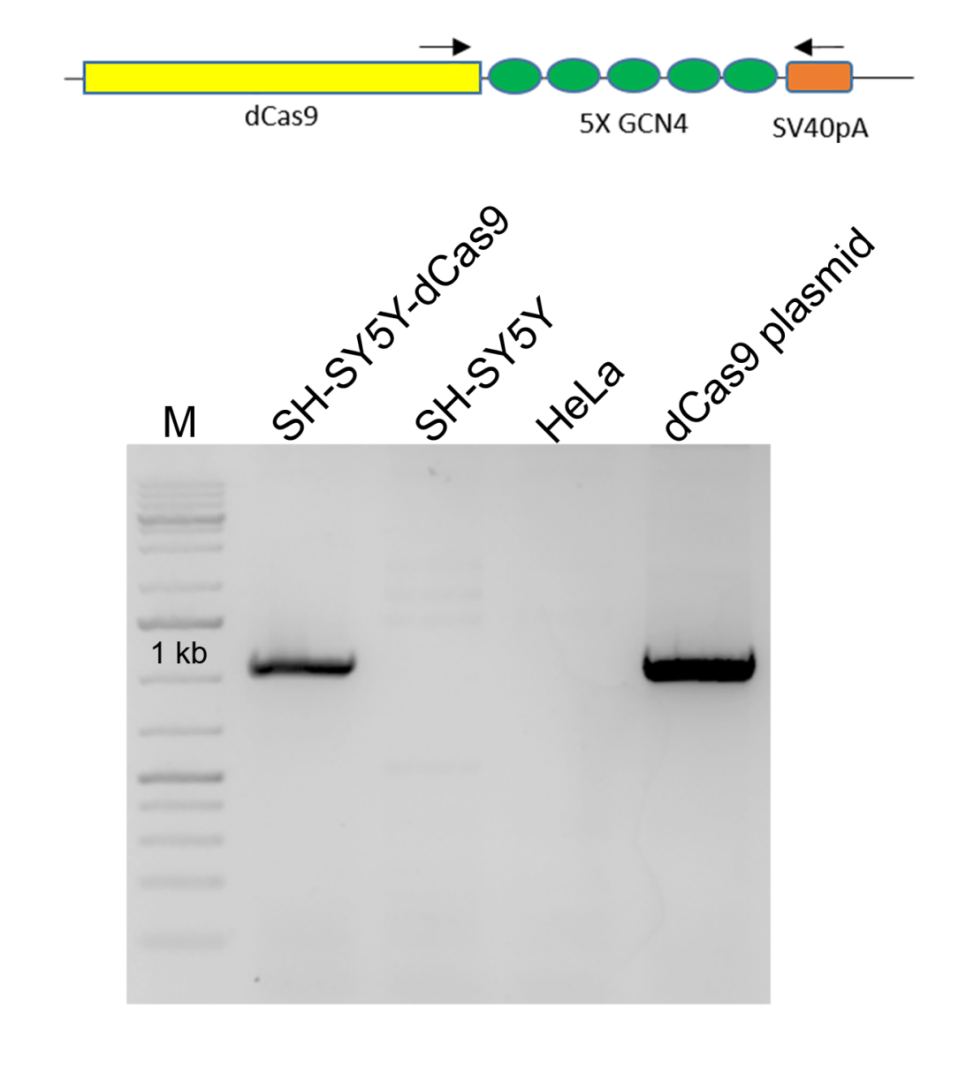
**

SH-SY5Y cells expressing dCas9-5xGCN4 were established by blasticidin selection. To verify the chromosomal integration of dCas9-5xGCN4, we performed PCR with forward and reverse primers just outside of dCas9-5xGCN4. The expected sized band of ~1.1 kb was amplified from the genomic DNA isolated from SH-SY5Y cells stably expressing dCas9-5xGCN4. Two negative control cell lines, wild-type SH-SY5Y and HeLa cells, were used. The original dCas9-5xGCN4 plasmid was used as a positive control.

**Appendix Figure S6. dCas9-5xGCN4 was precisely recruited at the *SNCA* promoter as directed by sgRNAs.**


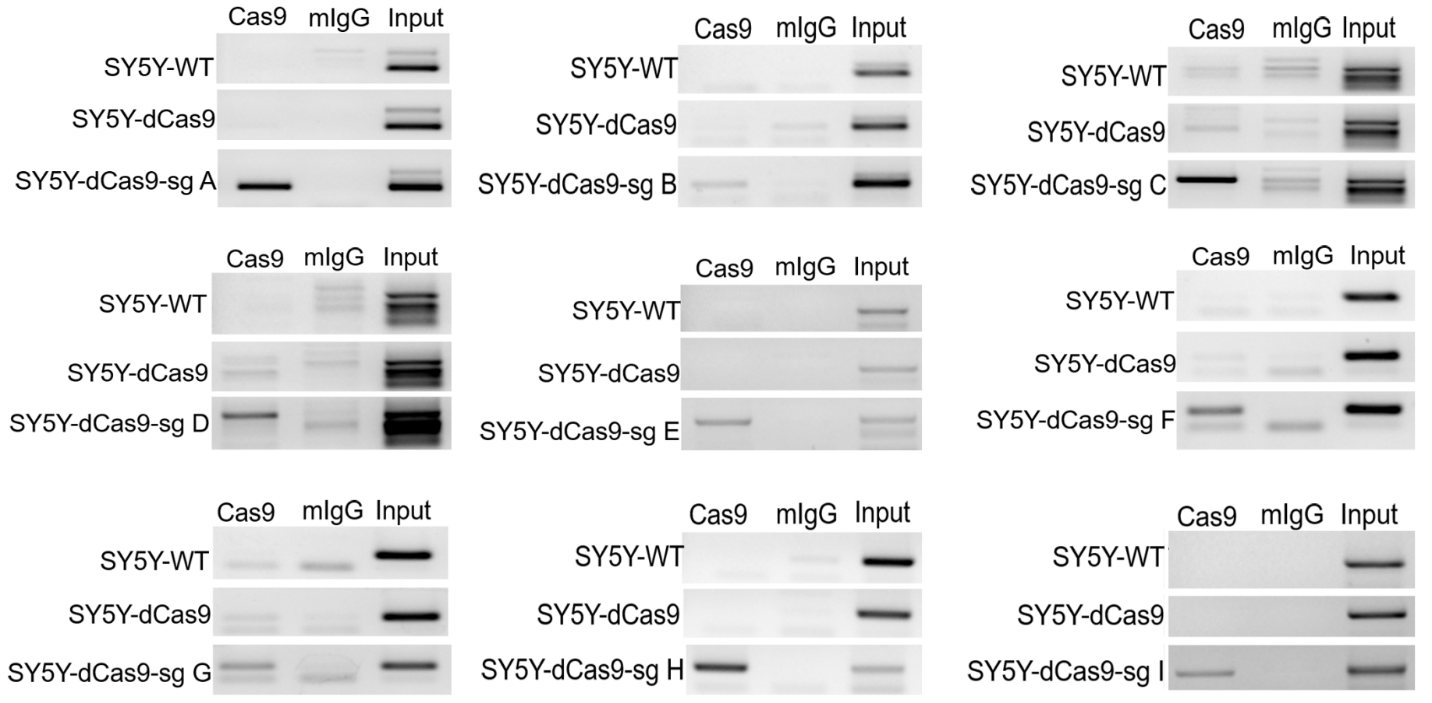


Each SH-SY5Y cell line stably expressing dCas9-5xGCN4 together with each sgRNA (A-I) was subjected to chromatin IP with cas9 antibody. Enrichment of dCas9-5xGCN4 was evaluated by PCR amplification using primer set encompassing the sgRNA binding sites. All the guide RNA sequences, corresponding primer sequences, and product sizes are listed in Appendix table 3. Wild-type SH-SY5Y cells and SH-SY5Y-dCas9-5xGCN4 were checked in parallel as negative controls. All nine sgRNAs successfully recruited dCas9-5xGCN4 at the site.

**Appendix Figure S7. Immunofluorescence staining and western blot images showing the presence of dCas9 in a representative sgRNA-dCas9 cell line.**


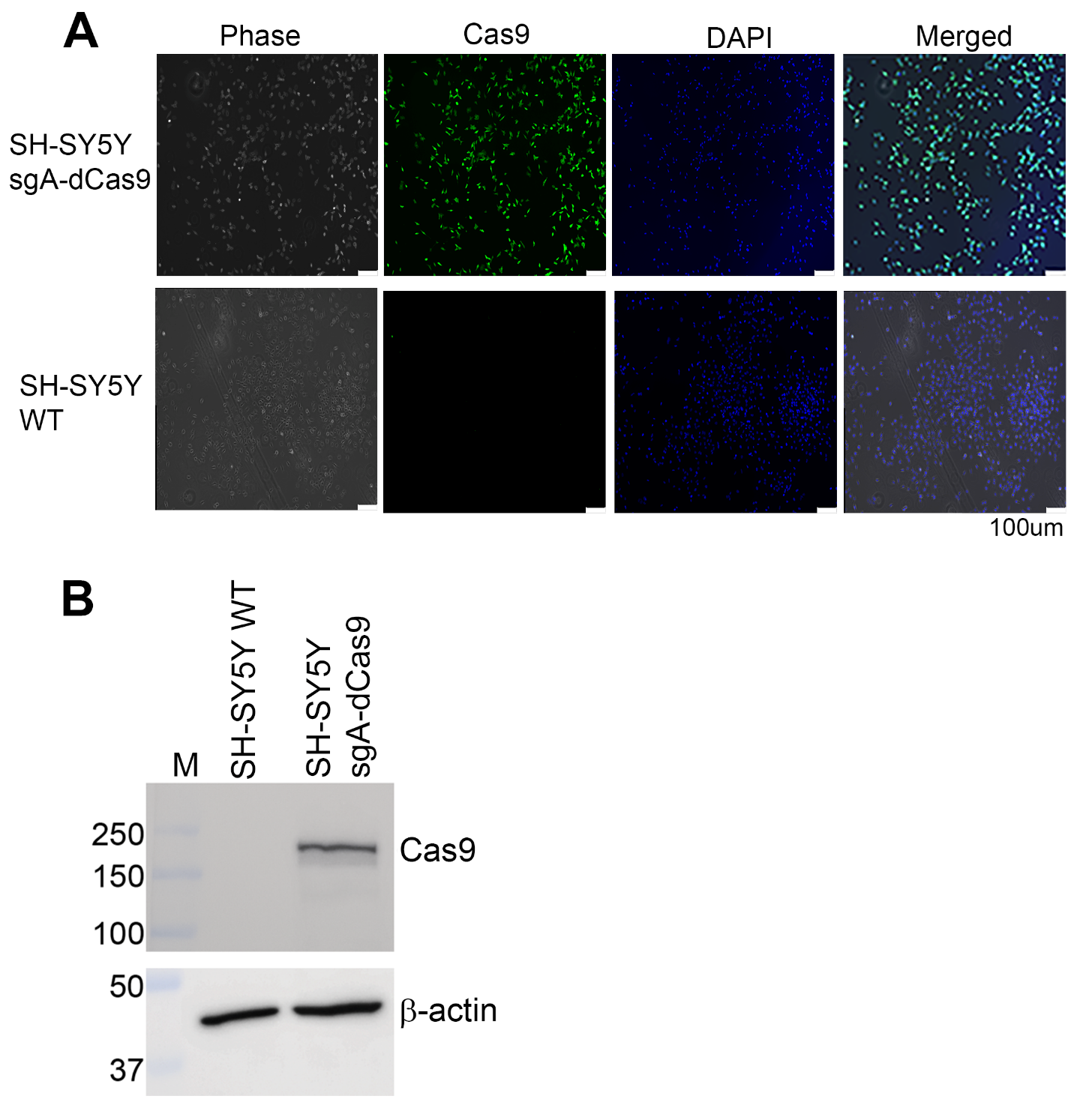


**(A)** Wild-type SH-SY5Y cell line and SH-SY5Y cells stably expressing sgA, dCas9-5X-GCN4 were immunostained with Cas9 (green). The cells were counter stained by DAPI and merged to show the presence of dCas9 only in the stably selected sgA-dCas9 lines but not in wild-type SH-SY5Y cells. Scale bar for the image is 100 µM.

**(B)** The total protein from the wild-type SH-SY5Y and stable SH-SY5Y cells expressing sgA-dCas9 were immunoblotted against Cas9 antibody. The stable SH-SY5Y-sgA-dCas9 cells only show the presence of Cas9, approximately at 168 kDa size. The left most lane in the gel shows the molecular weight marker. The respective level of β-actin is shown in the bottom panel of the gel.

**Appendix Figure S8. Western blot image showing relatively higher levels of α-SYN expression in SH-SY5Y cells compared to other neuronal cell lines.**


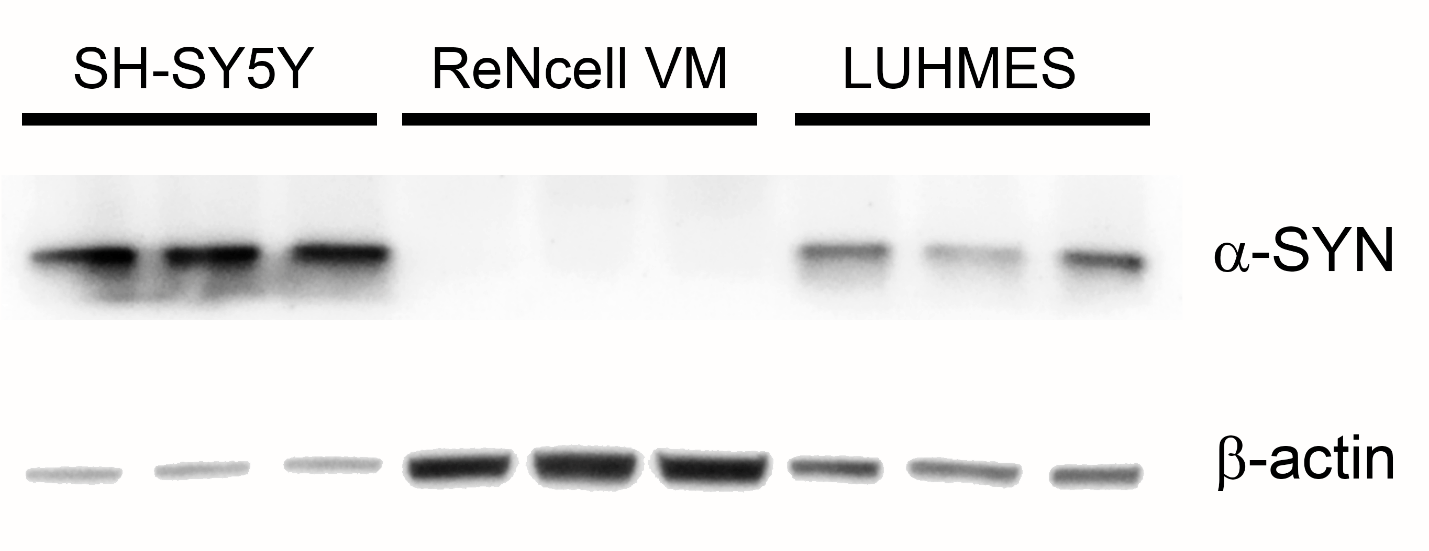


The total protein from three different neuronal cell lines SH-SY5Y, ReNcell VM and LUHMES were immunoblotted against α-SYN antibody. The respective β-actin levels are shown in the bottom panel. The experiment was performed in triplicate. SH-SY5Y cells show relatively higher levels of α-SYN as compared to the two other cell lines.

**Appendix Figure S9. Relative efficiency of sgRNAs in reducing H3K4me3 from the *SNCA* promoter.**

**
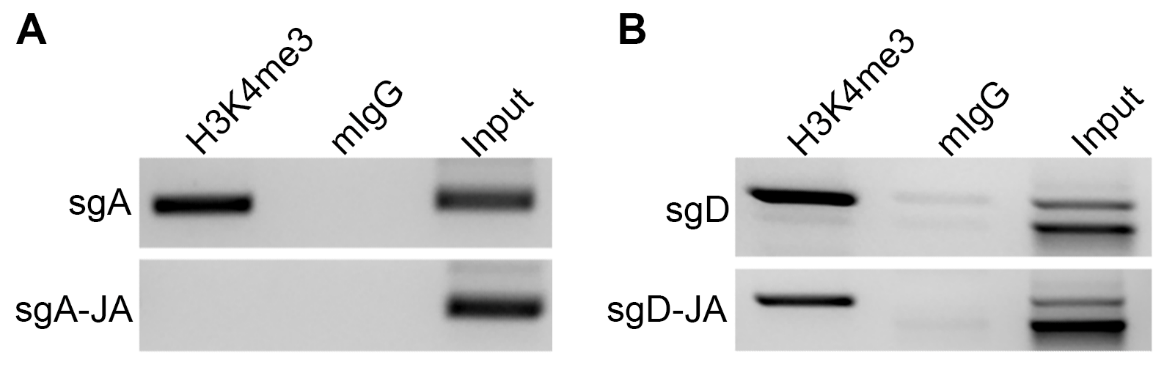
**

**(A, B)** ChIP gel images showing the enrichment of H3K4me3 between sgA and sgD. The efficiency of H3K4me3 reduction was compared in SH-SY5Y cells stably expressing sgA-dCas9-5xGCN4 (**A**) and sgD-dCas9-5xGCN4 (**B**) in the absence (sgA or sgD) or presence (sgA-JA or sgD-JA) of scFV-sfGFP-JARID1A. Results show sgA with JARID1A completely removed H3K4me3, while sgD showed partial reduction. Mouse IgG (mIgG) was used as an antibody control and 1% input was used as control for total chromatin.

**Appendix Figure S10. Fluorescence microscopy images showing sfGFP expression from a stable sgA-dCas9-JA cell line.**


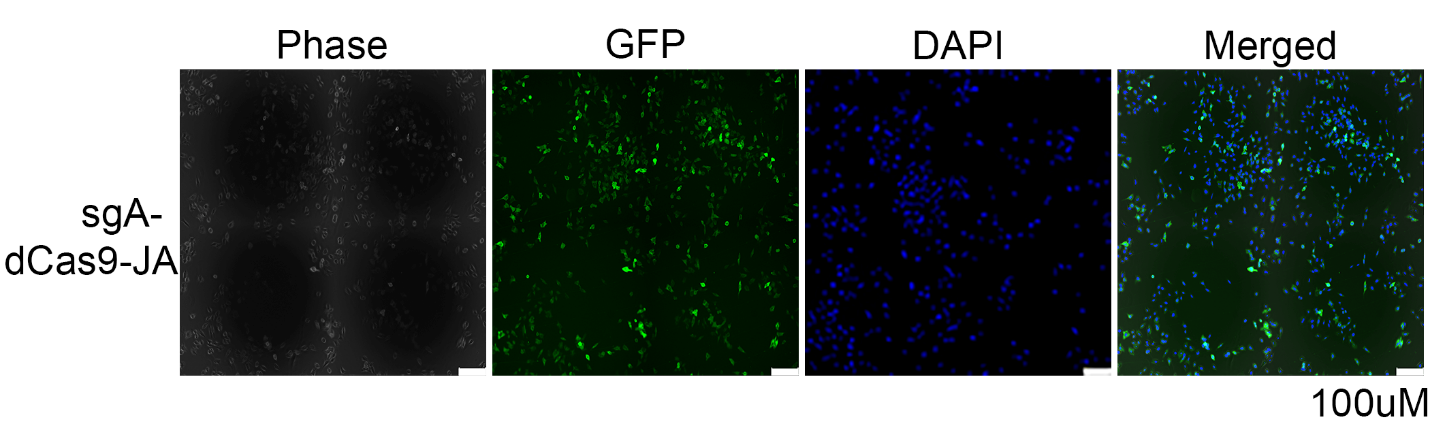


Stable SH-SY5Y cells expressing sgA-dCas9-JARID1A(JA), was evaluated for their GFP fluorescence originating from tagged sfGFP with scFv-JARID1A of the three-member transgene expression system. Cells were counterstained with DAPI and merged together with GFP. The scale bar of the image is 100 µM.

**Appendix Figure S11. Individual components of the CRISPR/dCas9 SunTag-JARID1A system do not affect α-SYN or global H3K4me3 levels.**


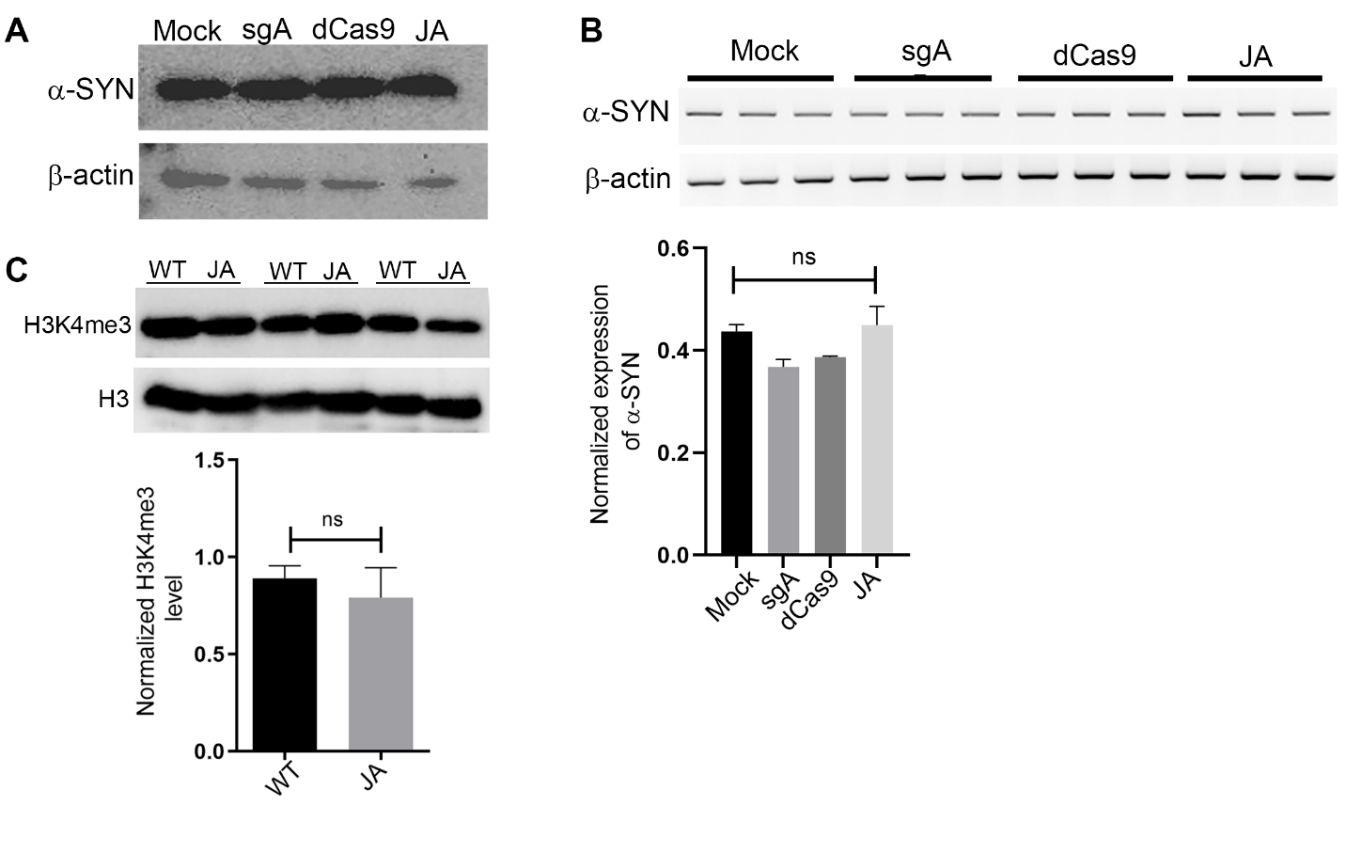


(**A**) The three components of the CRISPR/dCas9 SunTag-JARID1A system were individually overexpressed in SH-SY5Y cells. No difference in α-SYN expression levels was observed in western blots. β-actin was used as an internal control. (**B**) RT-PCR shows overexpression of the individual components of CRISPR/dCas9 SunTag-JARID1A system also did not significantly alter the expression of α-SYN. One-way ANOVA analysis was carried out to understand the difference between the group. (**C**) SH-SY5Y cell line stably expressing sgA-dCas9-JARID1A (JA), and wild-type cells (WT) were compared for global H3K4me3 levels. No significant difference in global H3K4me3 levels was observed. Total H3 level was used as an endogenous control. Three independent repeats were performed (n=3). Data are presented as mean ± SEM. ns, no significant difference in the mean. Data were analyzed using non-parametric t-test followed by Mann-Whitney post-hoc corrections. Two-tailed p-values was calculated.

**Appendix Figure S12. Additional gel pictures for Figure 5 and Figure 6.**

**
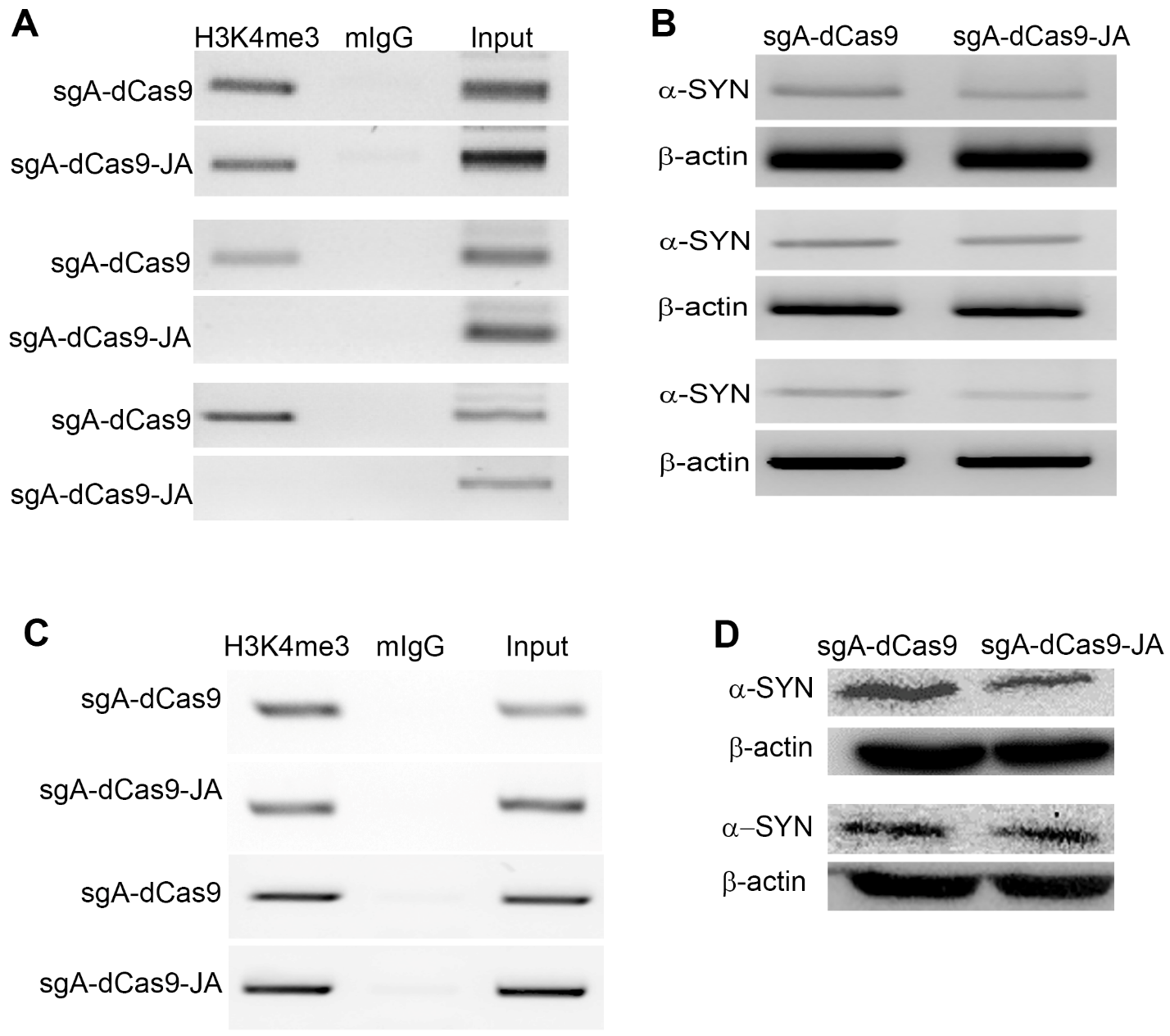
**

**(A)** Panel A shows the three more independent gel pictures for ChIP assay used in Figure 5A. (**B**) Panel B shows the three more independent RT-PCR gel pictures used in calculation for Figure 5B. (**C**) Panel C shows the two more sets of ChIP gel pictures used in the calculations for Figure 6E. (**D**) Panel D shows the two more sets of western blot images used in the calculations for Figure 6F.

**Appendix Figure S13. Characterization of iPSCs.**


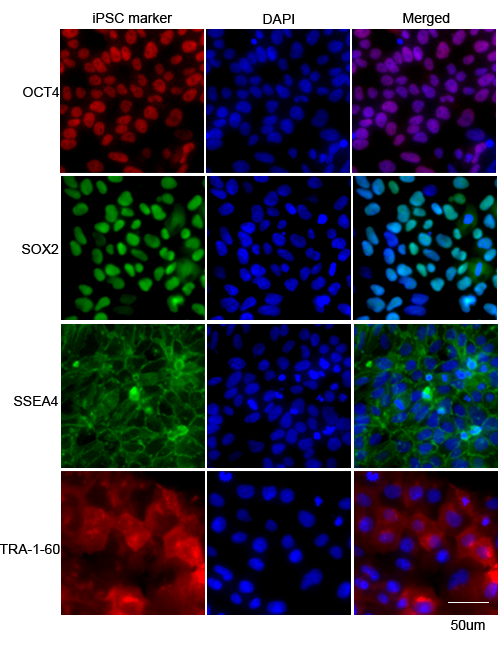


One of iPSC lines used in the study was evaluated for their stem cell characteristics. The cell line was stained for several pluripotency markers such as OCT4 (red; top panel), SOX2 (green; panel 2), SSEA-4 (green; panel 3) and TRA-1-60 (red; bottom panel). Cells for all markers were counter stained for DAPI. The merged images between respective marker and DAPI are shown on the right most quadrat of every image. The scale bar for this image is 50 µm.

**Appendix Figure S14. Immunostaining of differentiated sPD iPSCs demonstrates successful differentiation to dopaminergic neurons.**


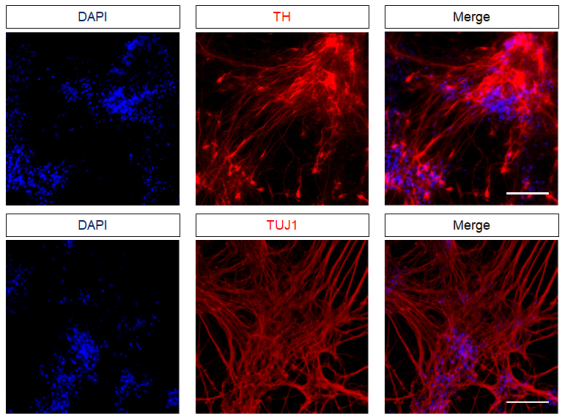
\

sPD1-1 line was differentiated for 30 days and subjected to immunostaining for the neuronal marker, β-III tubulin (TUJ1), and TH, a dopaminergic neuron marker. Majority of the cells expressed TUJ1 and TH, confirming adequate differentiation into dopaminergic neurons. Hoechst was used to stain the nuclei. Scale bar, 100 µm.

**Appendix Figure S15. Sequence of scFV-sfGFP**-**JARID1A construct in pLvx vector.**


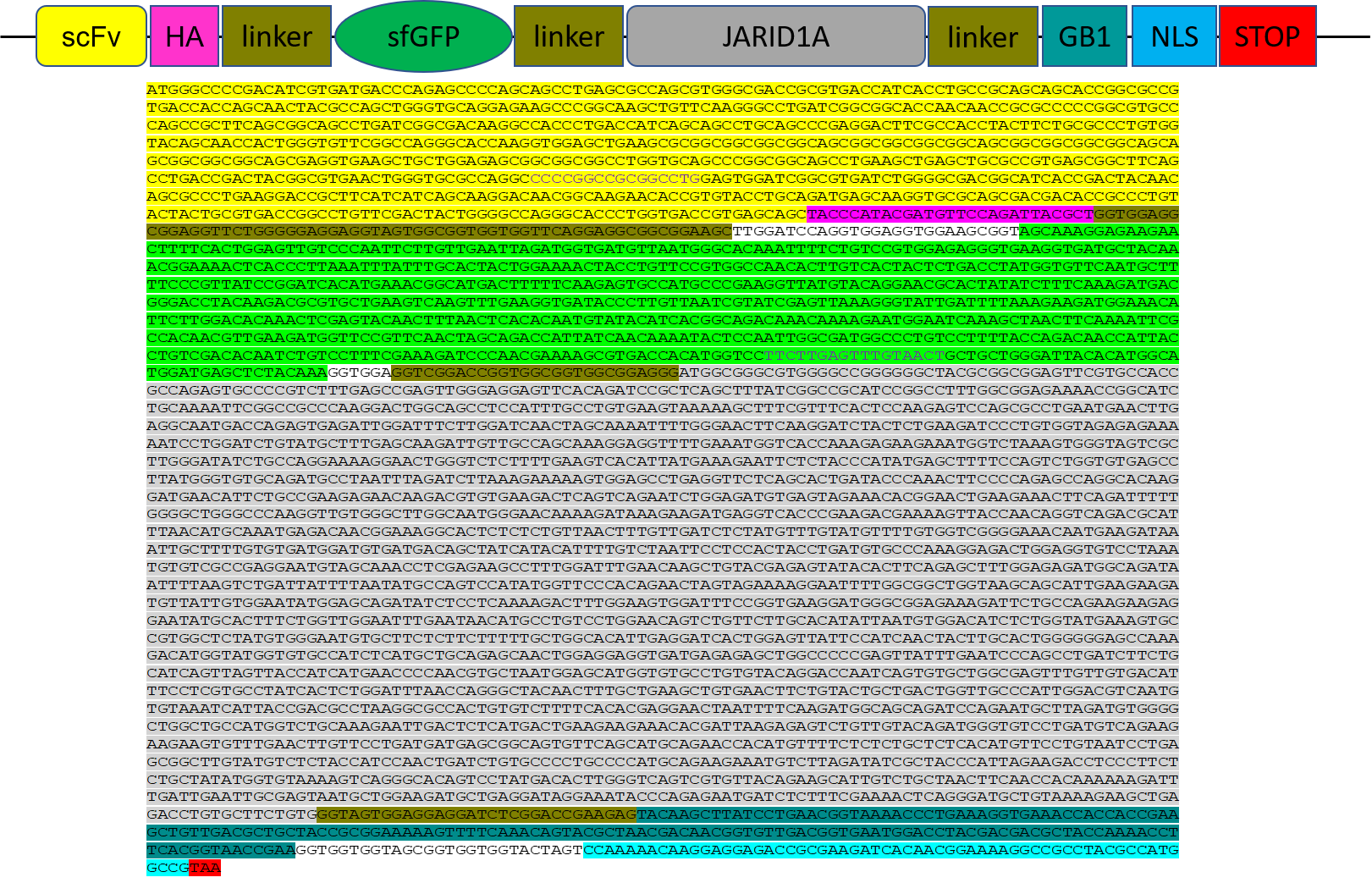


Organization of the scFV-sfGFP-JARID1A vector shown in Appendix Figure Sure 4. The sequence of the entire insert cloned in a pLvx-DsRed vector is shown. The sequence of each component of the vector is color-coded both in the diagram and in the sequence.

**Appendix Table S1. The details of post-mortem brain tissue samples used in the study.**

| **Source** | **Sample Number** | **Diagnosis** | **Age/Sex/ethnicity/race** | **Brain Region** | **PMI (h)** | **Braak staging** |
| --- | --- | --- | --- | --- | --- | --- |
| McLean Hospital, Harvard Medical School | 1 | PD | 70/M/NHL/W | SN | 26.36 | Early stage 4 |
| McLean Hospital, Harvard Medical School | 2 | PD | 79/M/NHL/W | SN | 10.1 | Early stage 5 |
| McLean Hospital, Harvard Medical School | 3 | PD | 89/M/NHL/W | SN | 19.92 | Stage 4 |
| McLean Hospital, Harvard Medical School | 4 | PD | 85/M/NHL/W | SN | 24.75 | Stage 4 |
| McLean Hospital, Harvard Medical School | 5 | PD | 87/M/NHL/W | SN | 24.72 | Stage 4 |
| McLean Hospital, Harvard Medical School | 6 | PD | 74/M/NHL/W | SN | 26.98 | Stage 5-6 |
| McLean Hospital, Harvard Medical School | 7 | PD | 89/M/NHL/W | SN | 29 | Early stage 4 |
| McLean Hospital, Harvard Medical School | 8 | PD | 74/M/NHL/W | SN | 35.42 | Stage 3 |
| McLean Hospital, Harvard Medical School | 9 | PD | 77/M/NHL/W | SN | 6.62 | Stage 4 |
| McLean Hospital, Harvard Medical School | 10 | PD | 71/M/NHL/W | SN | 15.67 | Early stage 4 |
| Human Brain and Spinal Fluid Resource Centre, Brain bank, UCLA | P1 | PD | 73/M/NHL/W | SN | 15 |  |
| Human Brain and Spinal Fluid Resource Centre, Brain bank, UCLA | P2 | PD | 79/M/NHL/W | SN | 12.3 |  |
| Human Brain and Spinal Fluid Resource Centre, Brain bank, UCLA | P3 | PD | 82/M/NHL/W | SN | 13.0 |  |
| Human Brain and Spinal Fluid Resource Centre, Brain bank, UCLA | P4 | PD | 83/M/NHL/W | SN | 6.7 |  |
| Human Brain and Spinal Fluid Resource Centre, Brain bank, UCLA | P5 | PD | 78/M/NHL/W | SN | 13 |  |
| Human Brain and Spinal Fluid Resource Centre, Brain bank, UCLA | P6 | PD | 87/M/NHL/W | SN | 11.2 |  |
| Human Brain and Spinal Fluid Resource Centre, Brain bank, UCLA | P7 | PD | 81/M/NHL/W | SN | 9.7 |  |
| Human Brain and Spinal Fluid Resource Centre, Brain bank, UCLA | P8 | PD | 75/M/NHL/W | SN | 13.8 |  |
| Human Brain and Spinal Fluid Resource Centre, Brain bank, UCLA | P9 | PD | 83/M/NHL/W | SN | 16.3 |  |
| Brain Endowment Bank, University of Miami, Miller School of Medicine | C1 | Control | 89/M/NHL/W | SN | 20.5 |  |
| Brain Endowment Bank, University of Miami, Miller School of Medicine | C2 | Control | 87/M/NHL/W | SN | 10 |  |
| Brain Endowment Bank, University of Miami, Miller School of Medicine | C3 | Control | 84/M/NHL/W | SN | 27.5 |  |
| Brain Endowment Bank, University of Miami, Miller School of Medicine | C4 | Control | 54/M/NHL/W | SN | 26.5 |  |
| Brain Endowment Bank, University of Miami, Miller School of Medicine | C5 | Control | 76/M/NHL/W | SN | 30.25 |  |
| Brain Endowment Bank, University of Miami, Miller School of Medicine | C6 | Control | 70/M/NHL/W | SN | 27.2 |  |
| Brain Endowment Bank, University of Miami, Miller School of Medicine | C7 | Control | 76/M/NHL/W | SN | 27.75 |  |
| Brain Endowment Bank, University of Miami, Miller School of Medicine | C8 | Control | 55/M/NHL/W | SN | 26.2 |  |
| Brain Endowment Bank, University of Miami, Miller School of Medicine | C9 | Control | 75/M/NHL/W | SN | 14.16 |  |

PD, Parkinson’s disease; NHL, Non-Hispanic or Latino; W, White

**Appendix Table S2. List of primers used in the study.**

| **Target** | **Experiment** | **5’-3’** | **Product size (bp)** |
| --- | --- | --- | --- |
| SNCA (promoter/intron1) | ChIP | F: TCCCCGGGAAACGCGAGGAT  R: CCCCGCGCCAGCACTTGTTA | 188 |
| SNCA (intron4) | ChIP | F:TGCCTTTGCATCAGATAATGGC  R:ATGATGAGCAGGCAGTCCG | 155 |
| α-synuclein | RNA | F:CACCATGGATGTATTCATGAA  R:AAAGATATTTCTTAGGCTTCAG | 437 |
| NeuN | RNA | F:CCCTTGCCGCTGGCTC  R:GCGACCACAGGAAGACTGTTA | 168 |
| Synpatophysin | RNA | F:TGCCAACAAGACCGAGAGTG  R:CAGAGCCCCCATGGAGTAGA | 196 |
| GFAP | RNA | F:GCACGCAGTATGAGGCAATG  R:TAGTCGTTGGCTTCGTGCTT | 139 |
| GAPDH | RNA | F:TTGCCATCAATGACCCCTTCA  R:TCCAAAATCAAGTGGGGCG | 153 |
| β-actin | RNA | F:GGAGTCCTGTGGCATCCACG  R:CTAGAAGCATTTGCGGTGGA | 322 |

**Appendix Table S3. List of short guide RNAs used in the study.**

| **Guide RNA** | **Sequence (5’-3’)** | **Validation primer sequence (5’-3’)** | **PCR product size (bp)** |
| --- | --- | --- | --- |
| sgA | AAGCAAAGGCTTTCTGCTAG  (sense) | F: AGCGCAAGAATCAGACAAAGC  R:AGAATGGAGAAGCAAGCTCCTC | 179 |
| sgB | TCCGGTAGGCTAAATCACGC  (sense) | F:AGTCAGAAAGGTGAGTGGTGTGTAG  R:AGCGGTCCTAAGGCTTTTCGCTCTAG | 164 |
| sgC | CCGCTTGTTTTAGACGGCTG  (sense) | F:AGTCAGAAAGGTGAGTGGTGTGTAG  R:AGCCGGAAAGGGTCCTGAGGG | 357 |
| sgD | TGGGAAAATCAGCGTCTGGC  (sense) | F:AGTCAGAAAGGTGAGTGGTGTGTAG  R:AGCCGGAAAGGGTCCTGAGGG | 357 |
| sgE | GCCGCGCAAGGCGGGAAAGT  (antisense) | F:AGCAGCTCCCCAAGGGATAGGCTC  R:CACGCACCTCACTTCCGCGT | 521 |
| sgF | AAGGGCAGACCAATAGTTCA  (sense) | F:CAAGGTCTCAAAGCCAGACAGCA  R:CTTGAACTATTGGTCTGCCCTTTGGA | 138 |
| sgG | CAAGTCCAACCTTCTTGCTC  (sense) | F:CAAGGTCTCAAAGCCAGACAGCA  R:CTTGAACTATTGGTCTGCCCTTTGGA | 138 |
| sgH | GCGACTCTGACGAGGGGTAG  (sense) | F:AGCAGCTCCCCAAGGGATAGGCTC  R:TGGAGATCGGGAGCGGTTGGGCTAG | 189 |
| sgI | ACTTTAAAACCACAAGGAAC  (antisense) | F:AGCAGCTCCCCAAGGGATAGGCTC  R:AGAATGGAGAAGCAAGCTCCTC | 521 |

**Appendix Table S4.** Exact p values in each figure.

| Figure number | Exact p value |
| --- | --- |
| Fig 1C | 3.37616E-08 |
| Fig 2B | 0.0510 |
| Fig 2C | 0.0129 |
| Fig 3D | 0.0134 |
| Fig 5A | 0.0424 |
| Fig 5B | 0.0286 |
| Fig 5C | 0.0037 |
| Fig 6C | 0.0260 |
| Fig 6D | 0.2684 |
| Fig 6E | 0.0500 |
| Fig 6F | 5.16634E-05 |
| Appendix Figure S2B | 0.7618 |
| Appendix Figure S2C | 0.0073 |
| Appendix Figure S3B | 0.1978 |
| Appendix Figure S11B | 0.0664 |
| Appendix Figure S11C | 0.5823 |
