# Supplementary figures and images for: Targeted attenuation of elevated histone marks at SNCA alleviates α‐synuclein in Parkinson's disease
Source: EMBO Mol Med. 2021 Jan 11;13(2):e12188. doi: 10.15252/emmm.202012188 (PMC7863397; doi:10.15252/emmm.202012188)

Figure 5B and Appendix Fig S12B

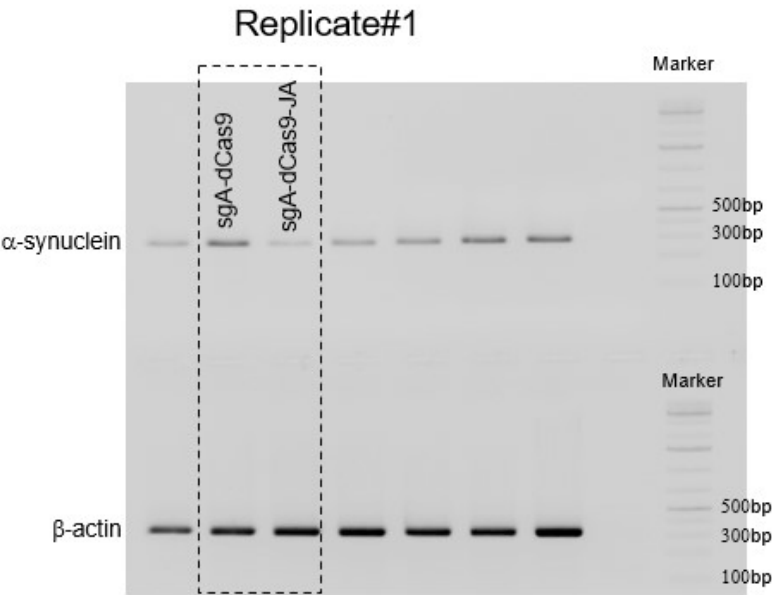

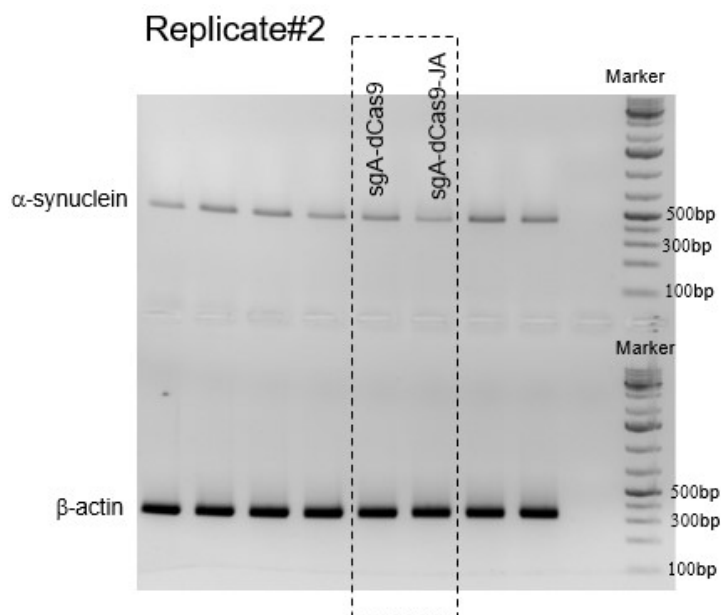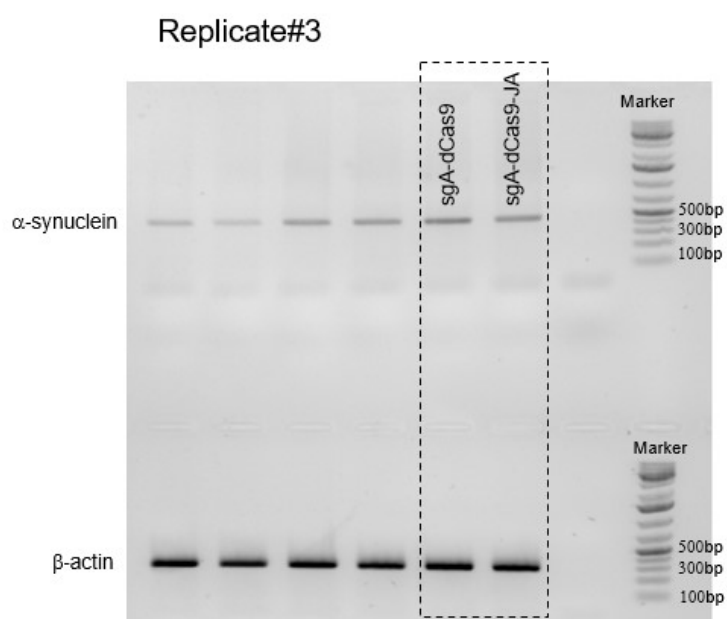

Replicate#4

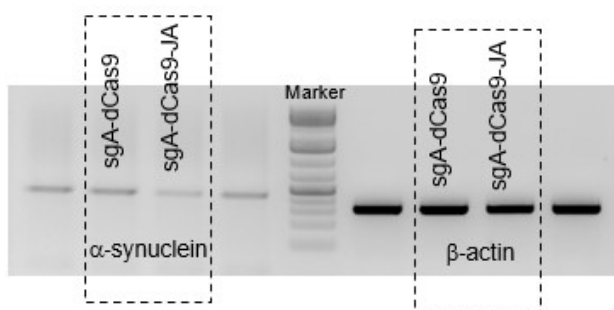

Supplement: Supplementary file 2 — Source Data for Appendix [file EMMM-13-e12188-s007.zip › SD for Appendix Figures/EMM_2020-12188_SourceDataFor_AppendixFigS12B.pdf]

Figure 5A and Appendix Fig S12A

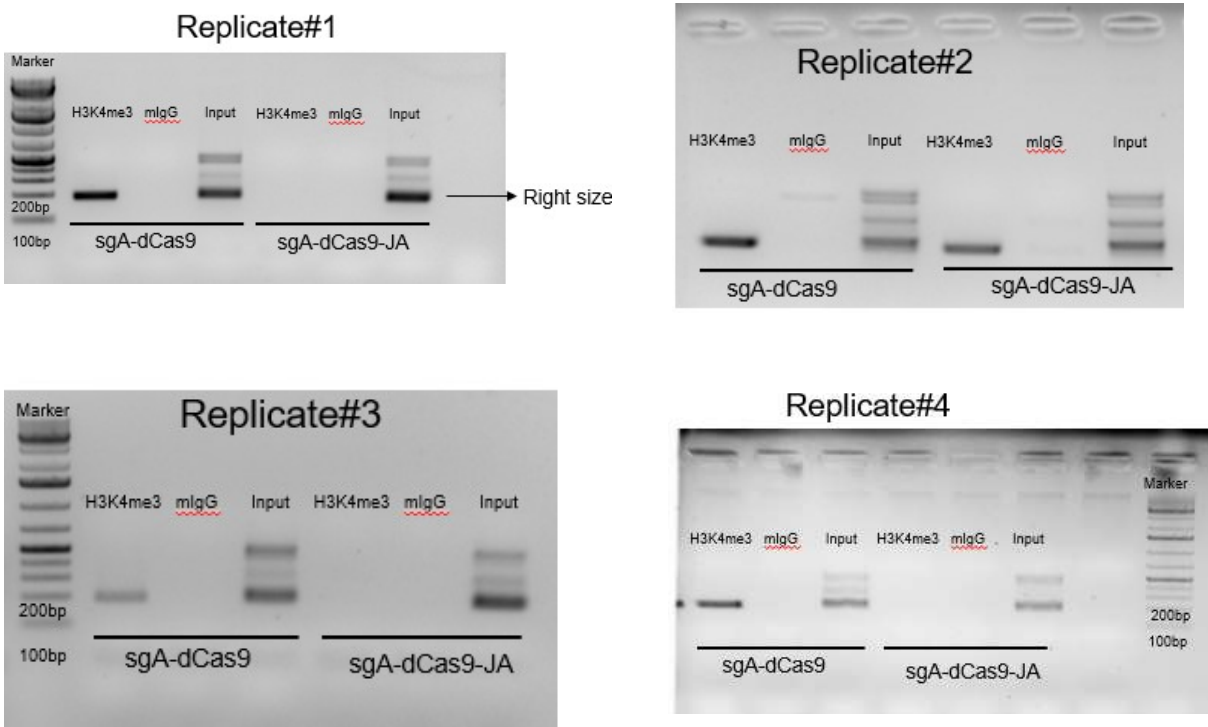

Supplement: Supplementary file 2 — Source Data for Appendix [file EMMM-13-e12188-s007.zip › SD for Appendix Figures/EMM-2020-12188_SourceDataFor_AppendixFigS12A.pdf]

Figure 6E and Appendix Fig S12C

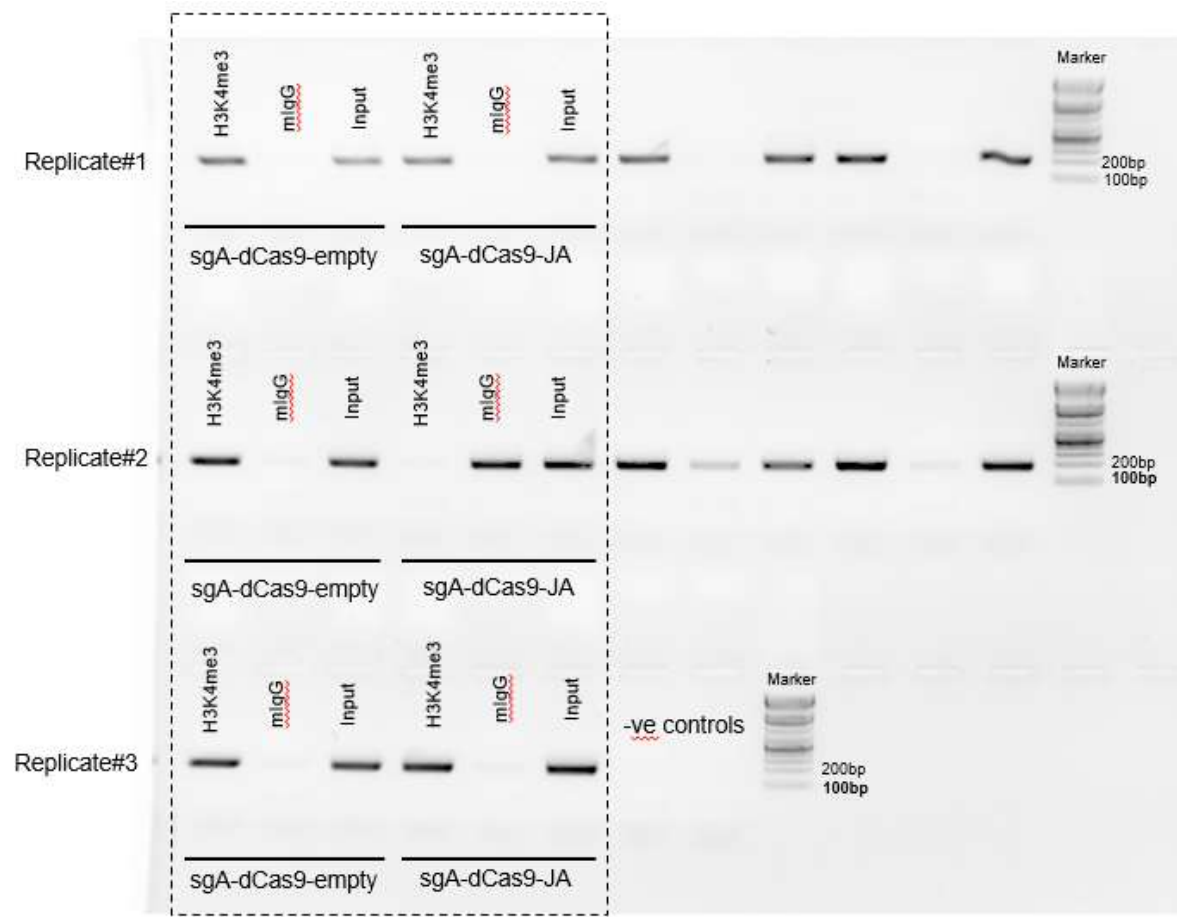

Supplement: Supplementary file 2 — Source Data for Appendix [file EMMM-13-e12188-s007.zip › SD for Appendix Figures/EMM-2020-12188_SourceDataFor_AppendixFigS12C.pdf]

**Appendix Fig S8**

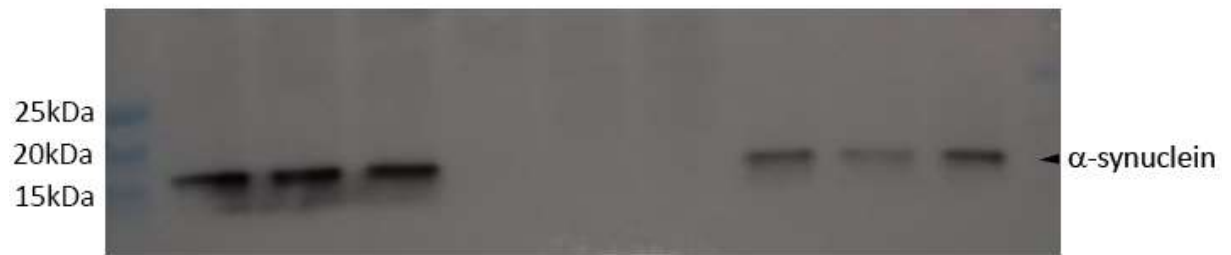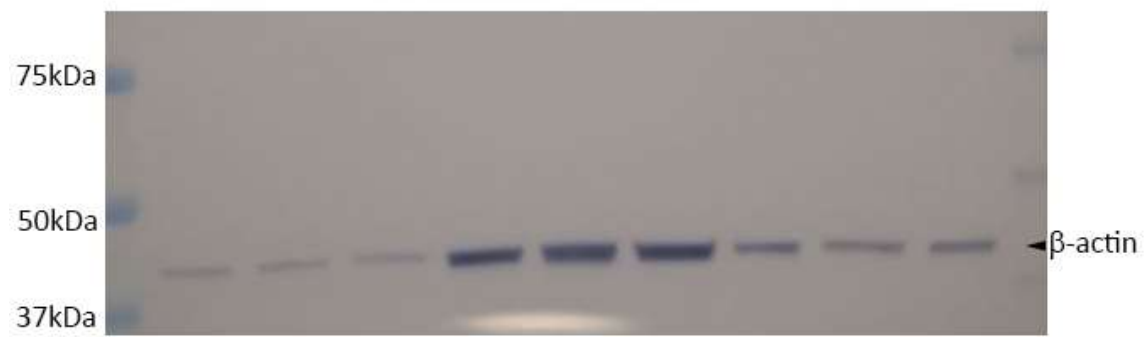

Supplement: Supplementary file 2 — Source Data for Appendix [file EMMM-13-e12188-s007.zip › SD for Appendix Figures/EMM-2020-12188_SourceDataFor_AppendixFigS8.pdf]

Figure 6F and Appendix Fig S12D

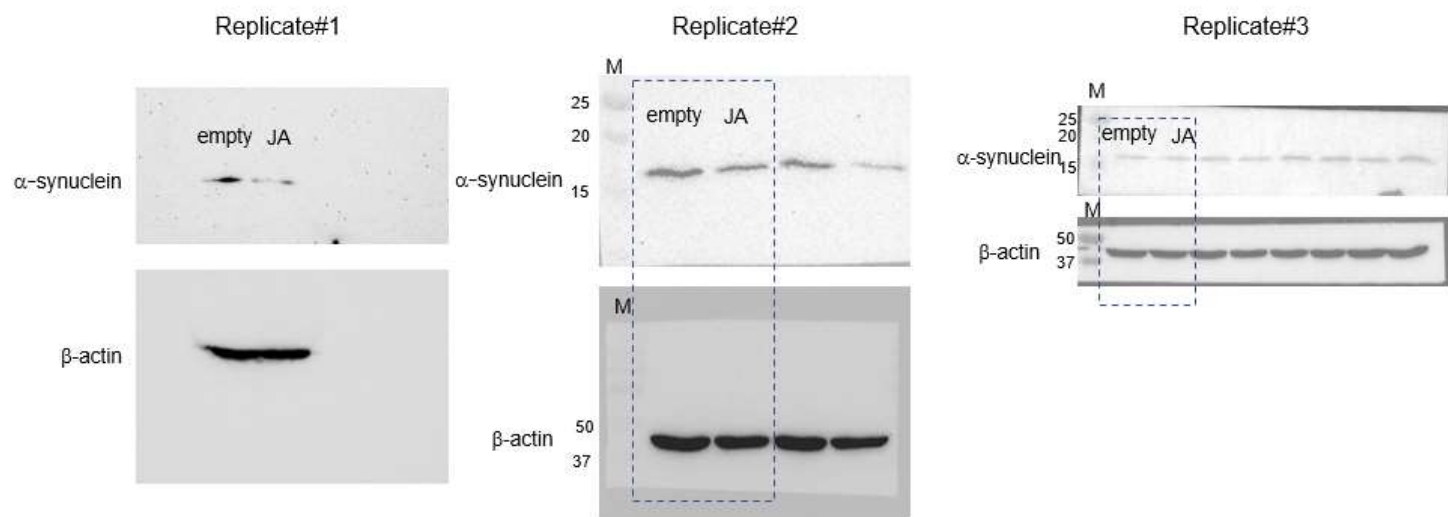

Supplement: Supplementary file 2 — Source Data for Appendix [file EMMM-13-e12188-s007.zip › SD for Appendix Figures/EMM-2020-12188_SourceDataFor_AppendixFigS12D.pdf]

Figure 1B

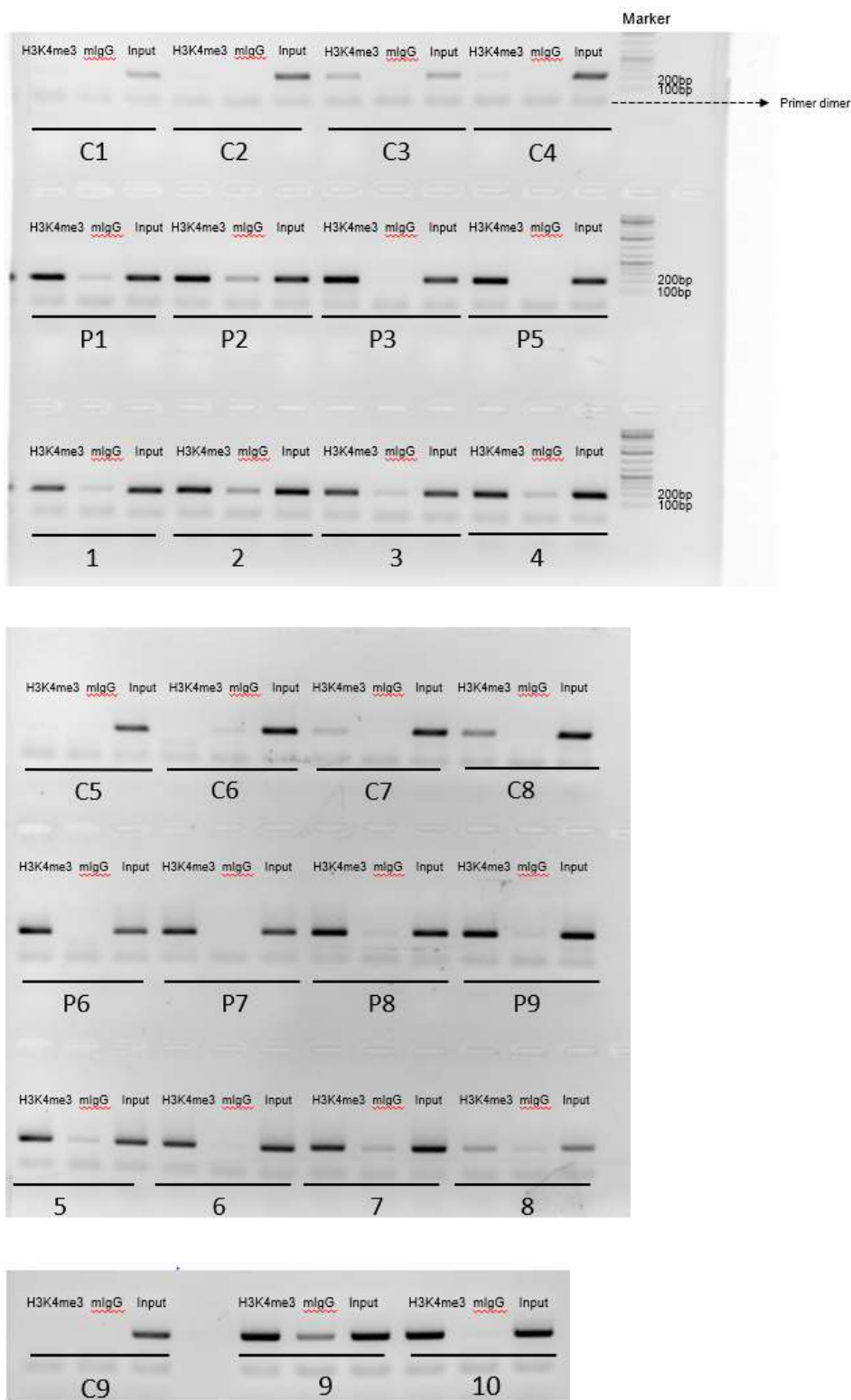

Supplement: Supplementary file 4 — Source Data for Figure 1 [file EMMM-13-e12188-s002.pdf]

Figure 3C

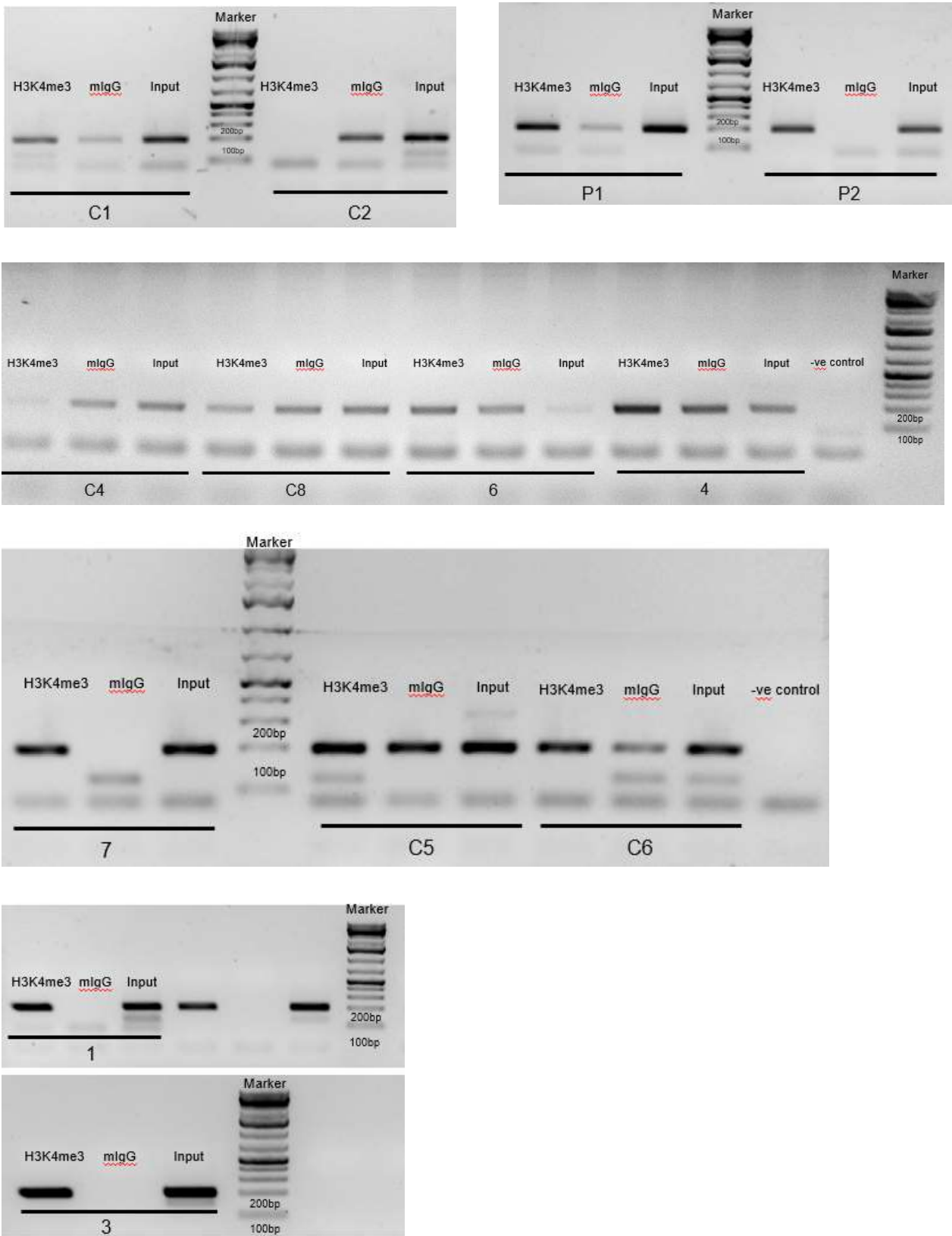

Supplement: Supplementary file 6 — Source Data for Figure 3 [file EMMM-13-e12188-s004.pdf]

**Figure 5C**

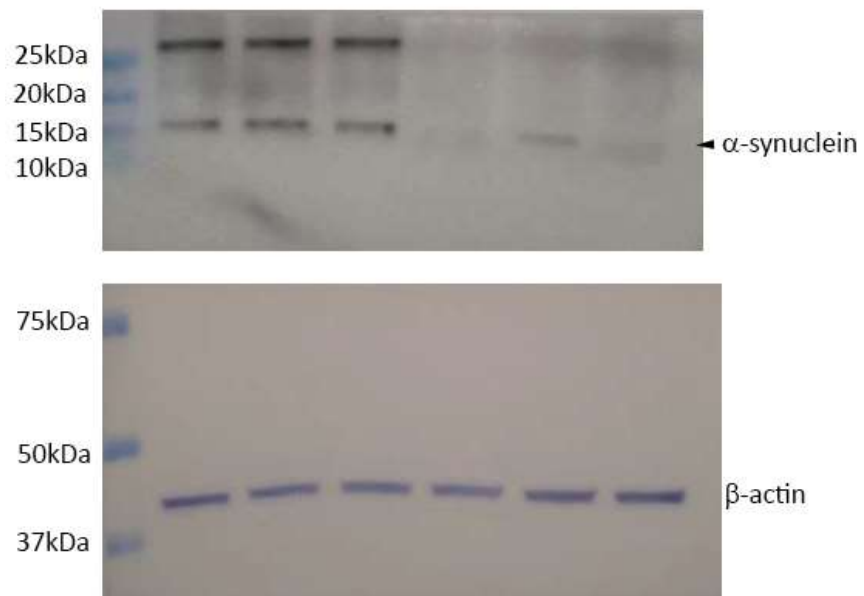

Supplement: Supplementary file 7 — Source Data for Figure 5 [file EMMM-13-e12188-s005.zip › EMM-2020-12188_SourceDataForFigure5C.pdf]

Figure 6A-B

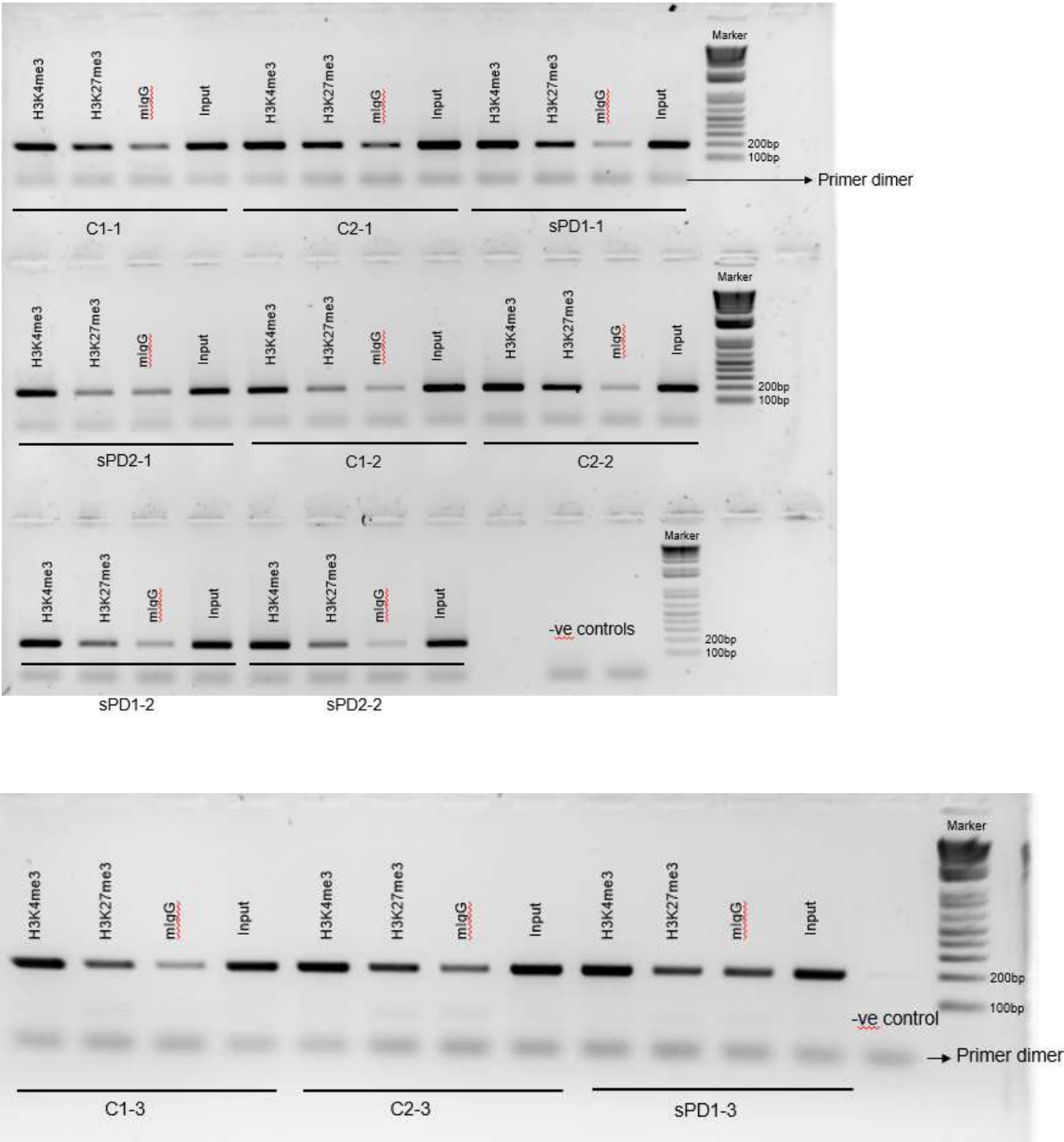

Supplement: Supplementary file 8 — Source Data for Figure 6 [file EMMM-13-e12188-s006.zip › EMM-2020-12188_SourceDataForFigure6A-B.pdf]
