# Supplementary material for: Targeted attenuation of elevated histone marks at SNCA alleviates α‐synuclein in Parkinson's disease
Source: EMBO Mol Med. 2021 Jan 11;13(2):e12188. doi: 10.15252/emmm.202012188 (PMC7863397; doi:10.15252/emmm.202012188)
Supplement: Supplementary file 5 — Source Data for Figure 2 [file EMMM-13-e12188-s003.pdf]

Figure 2A

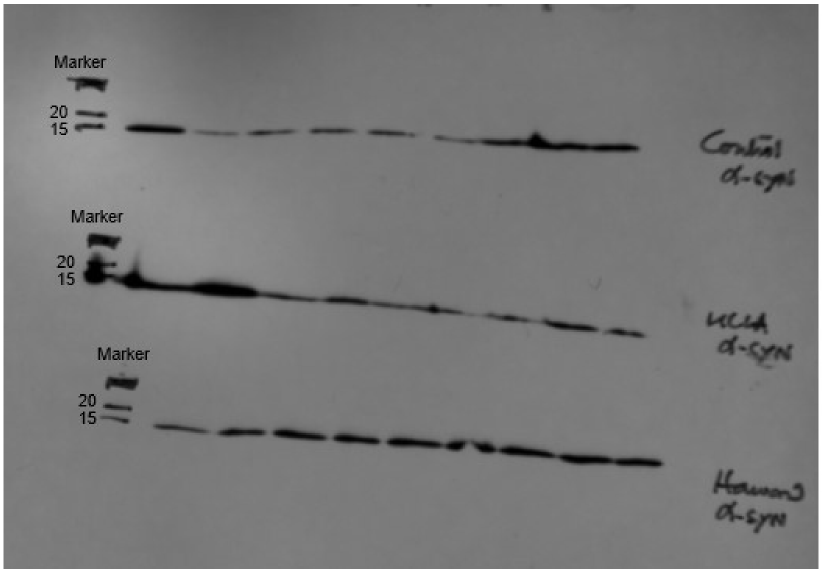

α-synuclein;
Samples C1-C9

α-synuclein;
Samples P1-P9

α-synuclein;
Samples 1-9

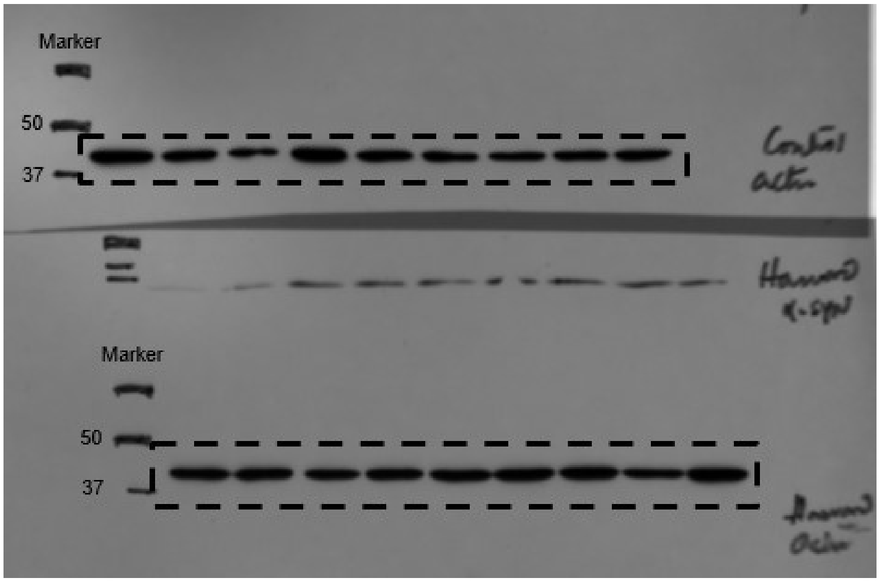

β-actin
SamplesC1-C9

β-actin
Samples 1-9

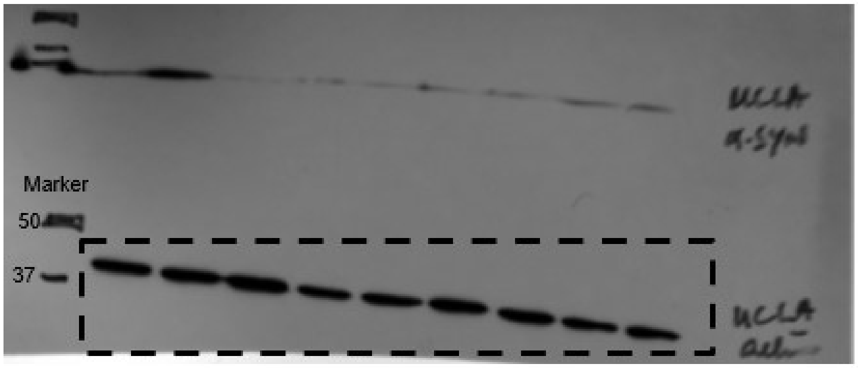

β-actin;
Samples P1-P9
